# Robotic assisted minimally invasive surgery

**DOI:** 10.4103/0972-9941.51313

**Published:** 2009

**Authors:** Jaydeep H Palep

**Affiliations:** Department of General Surgery, Grant Medical College and St. George's Hospital, Mumbai, India

**Keywords:** Robotic surgery, da Vinci surgery

## Abstract

The term “robot” was coined by the Czech playright Karel Capek in 1921 in his play Rossom's Universal Robots. The word “robot” is from the check word robota which means forced labor. The era of robots in surgery commenced in 1994 when the first AESOP (voice controlled camera holder) prototype robot was used clinically in 1993 and then marketed as the first surgical robot ever in 1994 by the US FDA. Since then many robot prototypes like the Endoassist (Armstrong Healthcare Ltd., High Wycombe, Buck, UK), FIPS endoarm (Karlsruhe Research Center, Karlsruhe, Germany) have been developed to add to the functions of the robot and try and increase its utility. Integrated Surgical Systems (now Intuitive Surgery, Inc.) redesigned the SRI Green Telepresence Surgery system and created the daVinci Surgical System^®^ classified as a master-slave surgical system. It uses true 3-D visualization and EndoWrist^®^. It was approved by FDA in July 2000 for general laparoscopic surgery, in November 2002 for mitral valve repair surgery. The da Vinci robot is currently being used in various fields such as urology, general surgery, gynecology, cardio-thoracic, pediatric and ENT surgery. It provides several advantages to conventional laparoscopy such as 3D vision, motion scaling, intuitive movements, visual immersion and tremor filtration. The advent of robotics has increased the use of minimally invasive surgery among laparoscopically naïve surgeons and expanded the repertoire of experienced surgeons to include more advanced and complex reconstructions.

## INTRODUCTION

The era of robots in surgery commenced in 1994 when the first AESOP (voice controlled camera holder) prototype robot was used clinically in 1993 and then marketed as the first surgical robot ever in 1994 by the US FDA^1^

Since then, many robot prototypes like the Endoassist (Armstrong Healthcare Ltd., High Wycombe, Buck, UK), FIPS endoarm (Karlsruhe Research Center, Karlsruhe, Germany) have been developed to add to the functions of the robot and try and increase its utility.

In 1997, Intuitive Surgicals Inc. (Menlo Park, Calif) came out with their prototype robot called the *da Vinci* which was a master-slave manipulator with three arms, one for the camera and two for operating the instruments. This has proved to be a breakthrough technology and stood the test of time since its inception.

### Ergonomic and technological comparison of *da Vinci* vis-à-vis conventional laparoscopy

Advanced laparoscopic surgery has a technically more demanding learning curve as against open surgery. The laparoscopic surgeon must view a distant monitor which provides 2-D vision, leading to a change in the normal hand-eye target axis.[1] The 2-D vision has a loss of stereoscopic depth perception which needs the surgeon to compensate for the same. Moreover, the camera is being held by an assistant and hence the vision is not under surgeon control and liable to fatigue, causing an unsteady field of vision.[2] All these factors lead to surgeon and assistant fatigue which are eliminated to a significant extent using the *da Vinci* surgical robot. The instruments in laparoscopic surgery are rigid and provide only four degrees of motion as compared to the surgical robot which provides seven degrees, just like the human wrist does, in open surgery. The abdominal wall also adds to this a ‘Fulcrum effect’ which reverses movements for the surgeon in laparoscopic surgery which is eliminated in robotic surgery just a in open surgery.[3] Hence, in conventional laparoscopy, tasks like ligation and suturing are much more complex.[4] All these factors of laparoscopy in colorectal surgery give a very prolonged and sustained learning curve.[5,6] However, the advantages of minimally invasive surgery are now confirmed beyond doubt with regard to oncological safety, survival and recurrence rates for malignant diseases.[7–13] The surgical robot can, therefore, be wisely used in choosing proper indications to provide the patient with the benefits of minimally invasive colorectal surgery eliminating the pitfalls of conventional laparoscopy at the same time.

### The *da Vinci* Surgical Robotic system

The *da Vinci* surgical robot is developed and marketed by Intuitive Surgical Inc. (Sunnyvale, CA). The first machine was setup in Europe in 1997 and the first surgical procedure was reported by Himpens *et al* in March 1997[14] Since its inception, the robot has been gradually upgraded from the first three-arm system to the current four arms, light weight and more versatile version called the S-Type. The system basically has three components: the robotic cart, the surgeon console and the endoscopic stack or column, details of which will be discussed subsequently. The system has technical features which significantly augment the quality and control of the visual field and thus enhance the dexterity of the surgeon. It delivers a high quality three dimensional (3-D) vision to the surgeon manning the console. This technology allows intuitive telemanipulation with tremor abolition, motion scaling and endo-wristed instruments. This is essentially what gives this technology an edge over the endoscopic technology which has been prevailing over the last two decades and overcomes some of the pitfalls of conventional laparoscopy which have probably limited the capabilities of the surgeon in the field of minimally invasive surgery.[15]

### Robotic cart

**Figure 1:**
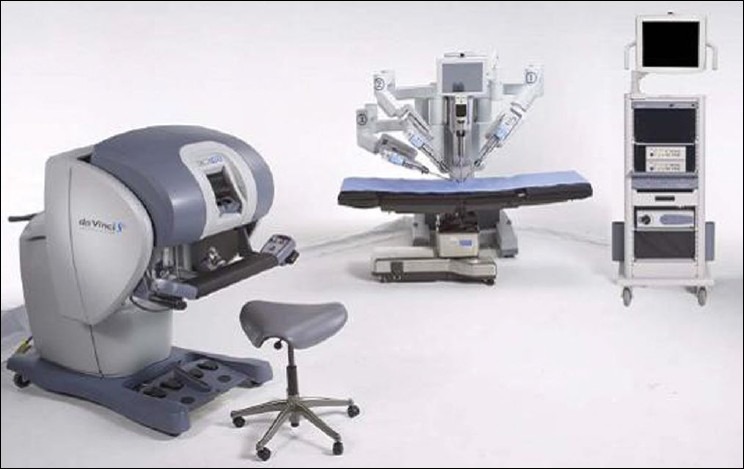
da Vinci S-Type (Intuitive Surgicals Inc., Sunnyvale, CA)

The robotic cart of the *S-Type da Vinci* [Figure 1] approximately 544 kg and is easily manoeuvrable on a wheel base. The cart is connected by color coded cables for the four arms to the console and the console in turn is connected the main power circuits. The cart consists of four robotic arms and a monitor for the assistant surgeon at the patient side. Once in position, the cart is locked in place slightly away from the operating table. The system runs off the main power system and has an emergency five-minute internal power backup.

Each arm has a series of multiple positioning joints and a terminal pivot joint at the attachment with the port allowing easy positioning of the arms during setup and a full range of movement during the surgery. Buttons provided at each joint allow manual adjustment by acting as a clutch, releasing the button locks the arm in place. The central, camera arm is compatible with a standard 12-mm port and the camera unit. The other three arms attach to specially designed 8-mm metal ports supplied with both blunt and sharp trocars. The arms are mechanically and electronically balanced for safety and ease of use. Custom-fitted plastic drapes are available to drape the four arms to achieve sterility, thus, allowing only the sterile ports and instruments in the operating field. The camera system (Insite vision system, Intuitive Surgical Inc.) has a dual lens system with two three-chip cameras and spatially separated within 12-mm casing. Hence, two complete optical systems are incorporated, representing the left and right eyes. The spatial separation of these images projected to the surgeon's eyes in the binocular viewer allows true 3-D image perception at the console. The head end of the cart is fitted with a HD monitor for the benefit of the assistant surgeon and the scrub nurse.

**Figure 2:**
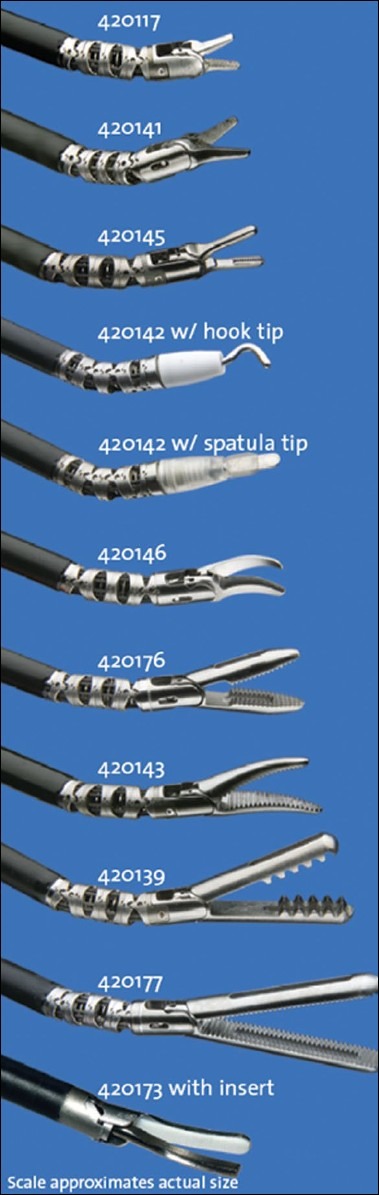
Instruments

**Figure 3:**
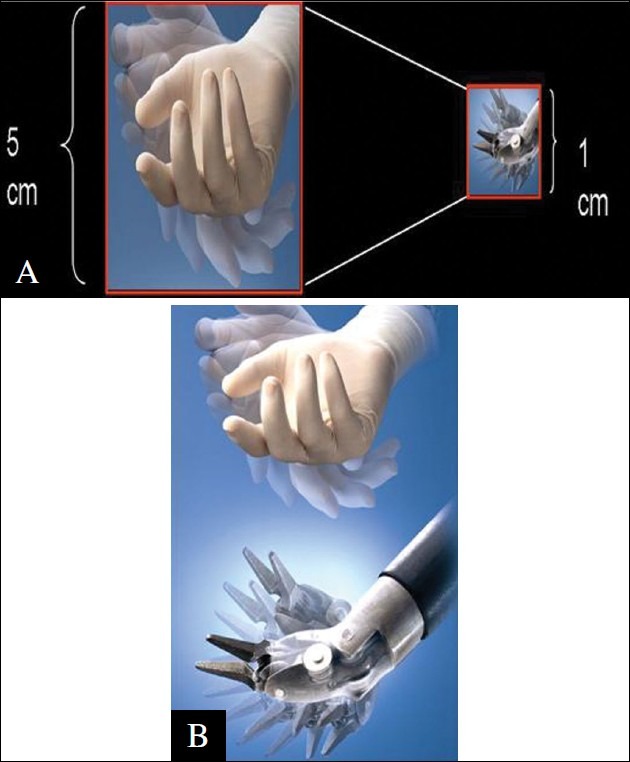
A. Endowrist instrument, B. Endowrist Instrument ergonomics

A wide variety of instruments [Figure 2] available with the system are easily and rapidly changeable by the assistant surgeon or a trained scrub nurse at the patient side. Except for the ultrasonic dissector all the instruments are endowristed (Endo Wrist, Intuitive Surgical Inc.). the instrument wrist is controlled by a cable system attached to four wheels on the instruments′ head that can be moved simultaneously by the robot to generate a single complex movement mimicking the motion of the human wrist [Figure 3]. The human tremors are effectively abolished by position sensing, which occurs approximately 1500 times per second. There are six degrees of motion at the instrument tip and a seventh degree of freedom provided by the instrument itself (e.g. grasping or cutting). Each instrument has only 10 lives following which it needs to be discarded and replaced this is done by the system which counts down the ten sessions of use. The instruments can be sterilized. The instrument can be used any number of times during one surgical procedure. However, a possibility of the instrument cable breaking off remains thus making the instrument unusable before 10 sessions.

### The Surgical Console

**Figure 4:**
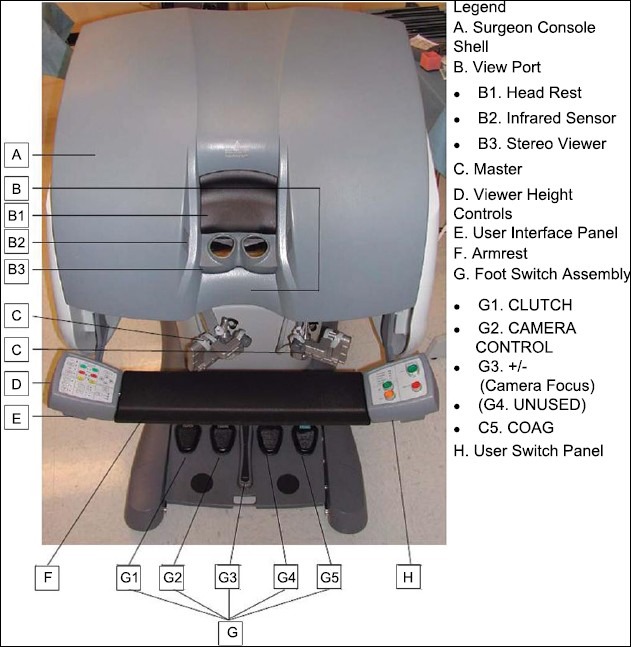
Surgeon console

This consists of the binocular viewer of the Insite vision system, the instrument controllers, the system setup and control panels, and five foot control pedals [Figure 4]. The console contains the hardware and the software of the computer which is essentially equivalent to 5 Pentium 300 processors (Intel Corp., Santa Clara, CA)

### Insite Vision System

**Figure 5:**
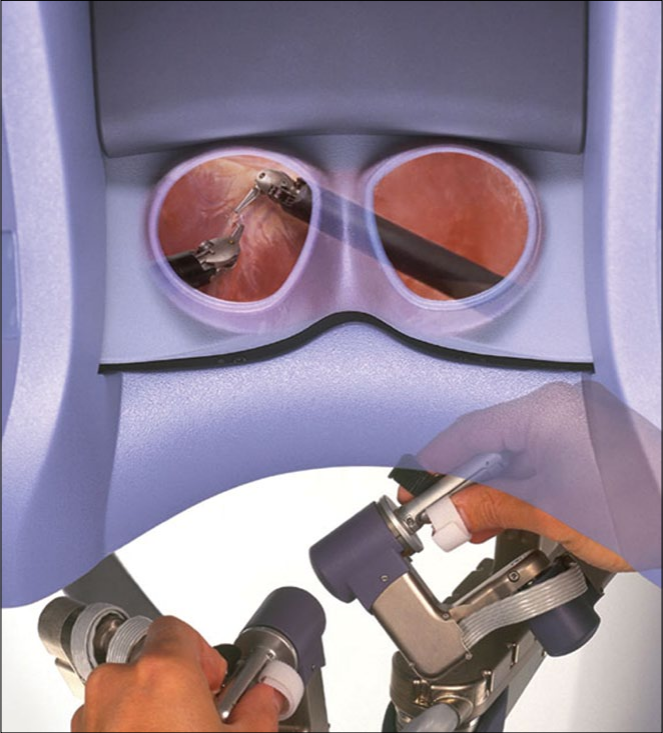
Binocular viewer

This is a high resolution endoscope, as mentioned earlier, consisting of two three-chip cameras and two optical channels generating two images delivered to each human eye viewing the binocular viewer [Figure 5]. Two light sources optimize the intensity of light. The surgeon controls image magnification by adjusting the depth of camera insertion in the operative field.

### Instrument control (Masters)

**Figure 6:**
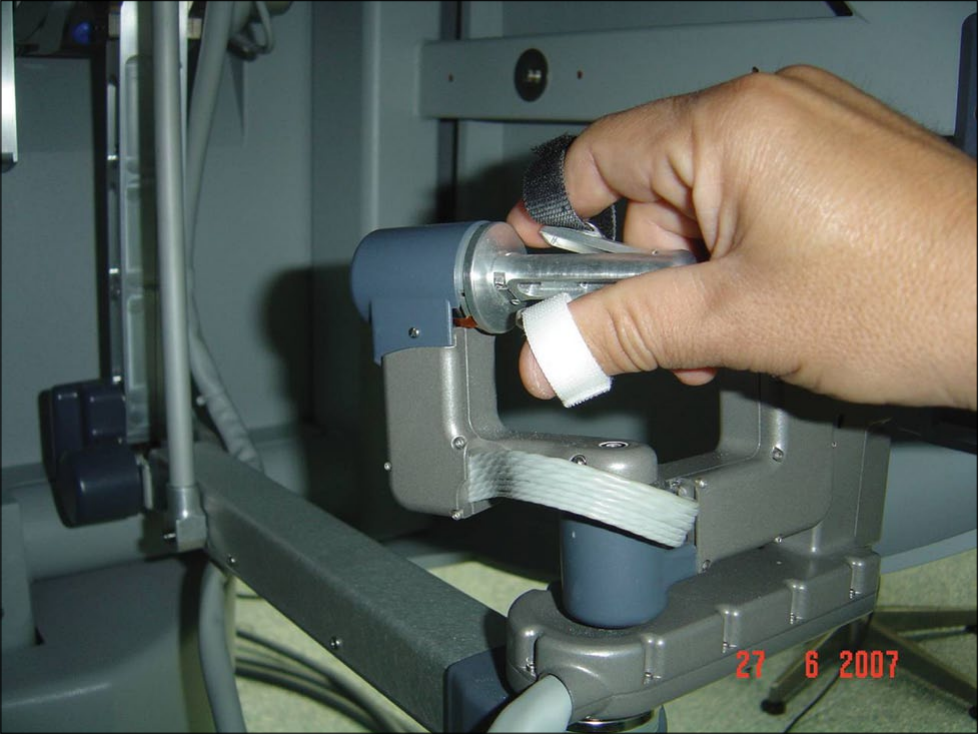
Masters

The surgeon is seated in an ergonomically comfortable position with the elbows resting on a padded bar. The thumb and index finger of each hand are placed in adjustable loops attached to the master controllers. Approximation of the thumb and index fingers operate the jawed instruments. The multi joint master controls move freely in all dimensions, allowing intuitive control of the instruments and the camera [Figure 6].

### Foot control

**Figure 7:**
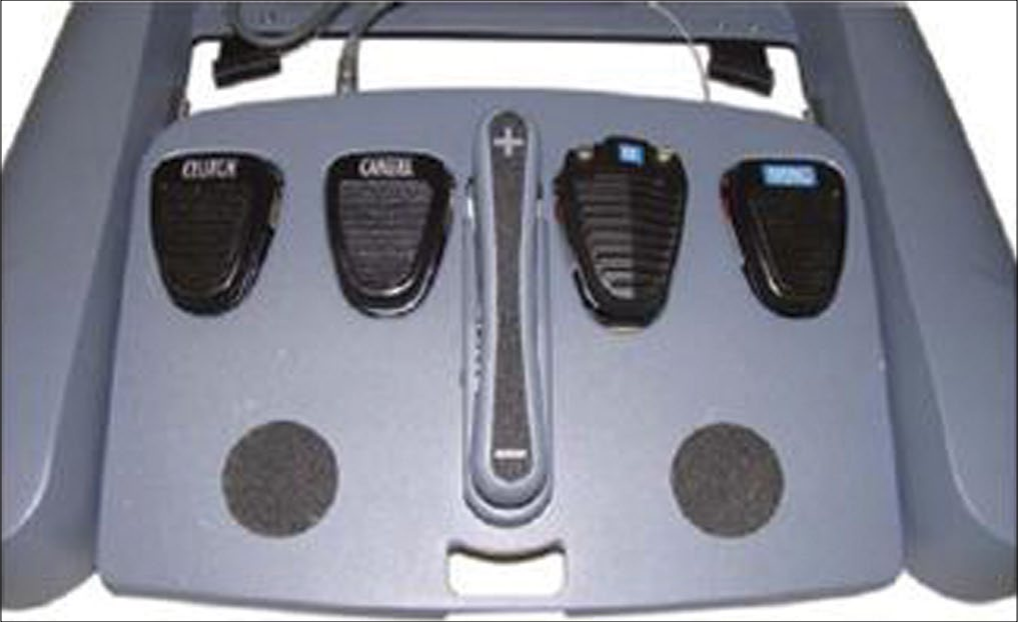
Foot control

There are five foot pedals. Starting from the left, the clutch pedal simply disengages the instruments from the controllers, allowing movement of the controllers to an ergonomically satisfactory position without changing the instrument position. The pedal to its right, i.e. the second one, is the camera pedal which when pressed disengages the instrument from the masters and engages the camera allowing adjustment of the camera depending on the need of the surgeon. The next foot switch adjusts focus and is used only initially before the commencement of the procedure normally to focus the telescope vision. The next foot switch is for bipolar coagulation and the fifth pedal is for monopolar cautery [Figure 7].

### Control panels

Two panels on either sides of the surgeon, one on the left includes camera and endoscopic calibration and motion scaling; the other on the right hand side controls the system start control, emergency stop control and standby buttons. Pressing the emergency stop button causes the master controllers to immediately disengage. This command can only be re-engaged by pressing the fault override button. In case of need to convert to open, the system can be rapidly disengaged by placing it on standby mode and disengaging the cart after removing the instruments and releasing the arms from the ports and the cart wheeled away from the field. Trained staff can normally achieve this in a matter of approximately two to four minutes.

### Ergonomics

The surgeon sits at the console with elbows resting on a padded bar, forehead placed against a padded bar with eyes comfortably viewing into the binocular viewer, the height of which is adjustable. The hand and finger positioning is as mentioned earlier. The intraocular distance can also be adjusted to suit the individual's needs. The ideal hand positions can be maintained, as the surgery progresses, by using the clutch pedal to reposition the surgeon's arms and hands.

### The Endoscopic stack [Figure 8]

**Figure 8:**
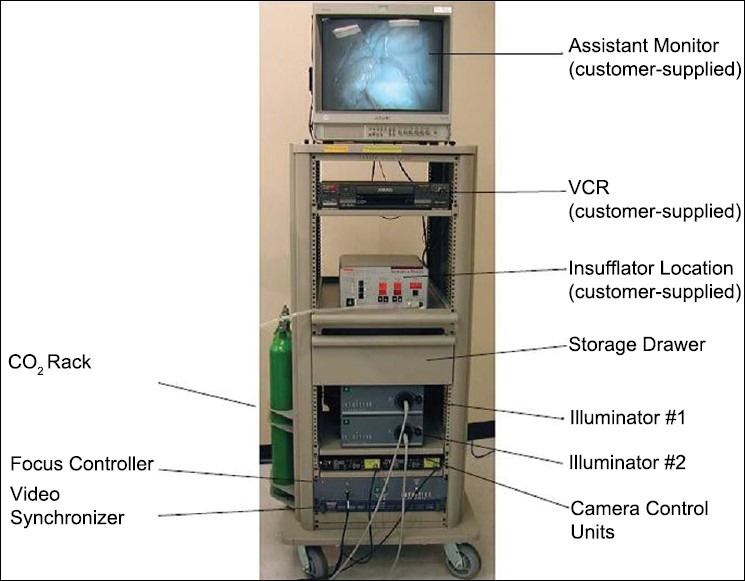
Endoscopic stack

This stack has all the features of a standard laparo / endoscopic stack viz: the monitor, a CO2 insufflator, a dual high intensity light source (Intuitive Surgical Inc.), and a dual CCD camera unit (Insite Vision, Intuitive Surgical Inc.). The features of the camera unit and light source are as already mentioned.

### Setup procedure

The robotic system start up sequence includes a self test that takes approximately one minute. The arms are draped thereafter, normally by two personnel, one being the scrub nurse and the other the OR assistant, who is not scrubbed, with practice, this usually takes less than 10 minutes. Once the camera and endoscope have been connected it needs to be calibrated. The system goes in to the standby mode by default. Now the patient has to be placed in the position desired and only then can the robotic ports be placed and the arms attached to start the procedure. Any movement of the operating table after the arms are fixed to the ports are contraindicated and can be extremely dangerous.

Once the arms are in place and the full range of movement of each arm is confirmed the desired instruments are placed by the scrub nurse or assistant surgeon through the ports in the operating field. The surgeon takes his position at the console and the ready button is pressed. As described earlier, the surgeon places his fingers in the adjustable loops and head in the binocular view and begins performing the surgery. An infrared sensor at the head pad engages the instruments and the camera just like pressing the ready button does.

The system is also incorporated with an audio intercom system which enables the surgeon's voice to be heard in the OR loud and clear, enabling the surgeon to keep looking in to the viewer and still talking to the assistants at the patient side. The other facilities included are an endoscopic cardiac stabilizer, ultrasonic instrumentation and the Gyrus plasma kinetic dissector. Needless to say, a large operating room is essential to house the robot and its components along with the routine equipment in the present day mini/invasive OR setup.

### Applications of the *da Vinci*

More than 1000 systems have now been setup across the globe, a majority of them being in the USA. The system was designed for use in minimally invasive surgery. However, it can be used for open surgery as well. The robot has been used until now mainly by urologists, general surgeons, cardiothoracic, gynaecologists and paediatric surgeons.

It is best suited for mini-invasive surgeries, especially those which are seemingly impossible or very difficult to perform with conventional laparoscopic techniques. Procedures like cholecystectomy, Nissens' fundoplication, adrenalectomy, rectopexy, cardiomyotomy, hernia repair and bariatric surgery were developed to be performed with standard instruments with the robotic technology.[15–22]

However, it is important to identify the right indications for the use of the robot simply because of the cost factor as of today. Hence, it is prudent to classify the indications as follows,

Surgeries improved with the robot- radical prostatectomy, radical cystectomy, pyeloplasty, partial nephrectomy, ureteric reimplantation, major hepatectomy, spleen preserving pancreatectomy, esophagectomy, gastric bypass, gastrectomy, nephrectomy, Heller's cardiomyotomy, pulmonary resections, rectal resection (with TME), difficult splenectomies.

Surgeries which can be performed only with the *da vinci* robotic system (vis-à-vis conventional laparoscopic surgery) - pancreatetoduodenctomies and other complex pancreatectomies, visceral artery aneurysmectomy, small sized hepatico-jejunostomy, microsutures (tubal anastomosis) and complex lymphadenectomies.[23]

To conclude, robotic surgery is an already well established technology being used across the globe. Presently the urologists and general surgeons are the frontrunners as far as the *da Vinci* system is concerned. Gynaecologists, paediatric surgeons, cardio-thoracic surgeons, ENT surgeons are taking the cue and incorporating it in their respective fields and the applications of the *da Vinci* are increasing by the day. The learning curve for advanced abdominal minimally invasive surgeries is sustained and long but achievable at specialised centers with a high volume of cases.

Any new therapeutic innovation is critical to our future health and such an innovation will, at least initially, cost more than the previous therapy. To abandon the search for improved therapies on the basis of cost would represent enormous disservice to our patients and would distinguish attempts to improve patient care from the quest for better automobiles, audio systems, or computers, or from any area of human endeavor.[24]
